# Thicker polyethylene inserts (≥ 13 mm) increase the risk for early failure after primary cruciate-retaining total knee arthroplasty (TKA): a single-centre study of 7643 TKAs

**DOI:** 10.1007/s00167-022-07189-8

**Published:** 2022-10-07

**Authors:** Anni Rajamäki, Mika Niemeläinen, Mika Junnila, Lari Lehtovirta, Mari Karsikas, Ville Ponkilainen, Antti Eskelinen

**Affiliations:** 1 Coxa Hospital for Joint Replacement, Niveltie 4, 33520 Tampere, Finland; 2 Department of Surgery, Central Finland Central Hospital Nova, Hoitajantie 3, 40620 Jyväskylä, Finland

**Keywords:** TKA, Polyethylene insert, Thickness, Revision risk, Instability, Cruciate-retaining implant, Survival, Aseptic loosening

## Abstract

**Purpose:**

This study investigates whether thicker (PE) inserts lead to a greater risk for revision after TKA. The differences between the TKA designs of three manufacturers (NexGen, PFC Sigma, Triathlon) are also compared.

**Methods:**

A total of 7643 primary TKA surgeries were included. PE inserts were divided into two groups—“thick PE inserts” with a thickness of 13 mm (mm) or more and “standard PE inserts” with a thickness of less than 13 mm. Three cruciate-retaining (CR) TKA designs (NexGen, PFC Sigma, Triathlon) were included in the study. The differences in failure rates between groups were investigated using Kaplan–Meier survival curves and Cox regression model with hazard ratios (HR). Failure rates were investigated short-term (< 2 years) and long-term (the whole follow-up period). The TKA designs were analysed both together and separately.

**Results:**

During the whole follow-up period, there were 184 (2.4%) aseptic revisions. The thick PE insert group showed an increased risk for revision compared to the standard PE insert group in both short-term (< 2 years; HR 2.0, CI 1.3 to 3.2) and long term (> 2 years; HR 1.6, CI 1.1 to 2.3) follow-up. The highest revision rate was observed in patients who received the Triathlon TKA with a thicker PE insert (HR 2.6, CI 1.2 to 5.7).

**Conclusion:**

The results indicate that thicker PE inserts are associated with increased risk for revision in primary TKA. Further research is required to ascertain whether more conformed PE inserts or constrained knee designs instead of thick CR inserts will ultimately lead to better clinical outcomes.

**Level of evidence:**

III.

**Supplementary Information:**

The online version contains supplementary material available at 10.1007/s00167-022-07189-8.

## Introduction

Using an appropriately sized polyethylene (PE) insert is an essential part of achieving good knee stability in contemporary primary TKA [1, 7, 11]. The most commonly used thicknesses of PE inserts range from 9 to 12 mm, but both thinner and thicker inserts are available for all contemporary primary TKA designs [6]. Choosing the correct thickness of PE insert is multifactorial [9]. In primary TKA, a thick (usually 13 mm or more) PE insert is occasionally needed if proper ligament stability is not otherwise achieved. Such situations can be caused by deep tibial resection, general ligamentous laxity, extension-flexion gap imbalance or iatrogenic collateral injury caused by the surgeon [2]. In such circumstances, using a thicker PE insert may not correct instability or imbalance. Moreover, if other surgical solutions to correct the situation are not used intraoperatively, the patient may require early revision surgery due to instability and residual knee symptoms. One reason for residual knee symptoms is patella baja, which is caused when a thicker PE insert elevates the joint line [3].

There are only three previous studies that have assessed the effect of PE insert thickness on risk for revision after primary TKA. The results of these studies are, however, contradictory. Furthermore, the threshold values used for the thick PE inserts in these studies were for extremely thick PE inserts (15–16 mm) and the sample sizes for the thick PE group remained small. There are, therefore, no previous studies where the threshold value is set low enough to make the results more practical for clinical work. In addition, no early revisions were studied separately, which could suggest more technical problems in TKAs [2, 4, 8].

In the current study, the aim was to investigate whether thicker polyethylene inserts lead to higher failure rates in primary TKA. The risk was also assessed for early failure separately. A secondary aim was to compare the revision rates of the cruciate-retaining implant designs of three different manufacturers. The hypothesis was that thicker polyethylene inserts would lead to higher failure rates.

## Materials and methods

**Fig. 1:**
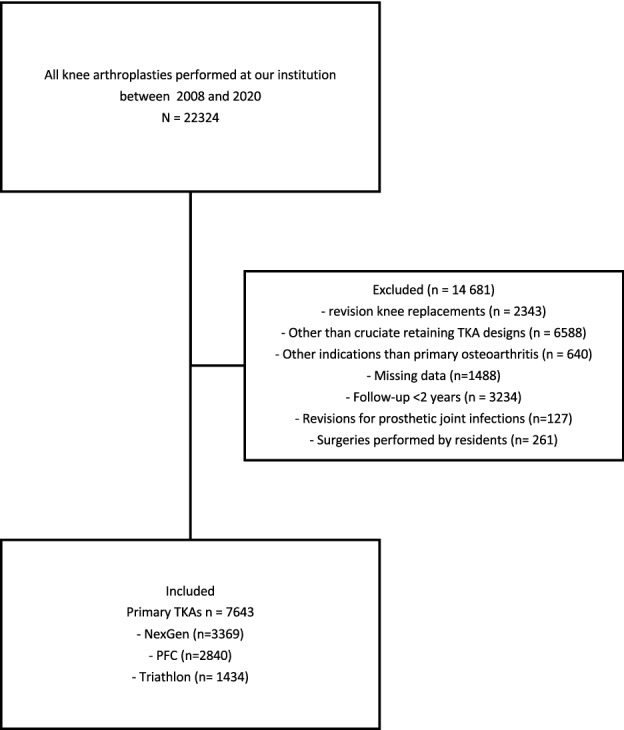
Flowchart of the formation of the patient cohort and reasons for exclusion

This study is a retrospective cohort study of all primary TKAs performed at a single institution between 1st January 2008 and 26th September 2020 that met the inclusion criteria. The three most frequently used implants at our institution, the NexGen (Zimmer Biomet, Warsaw, In), the PFC Sigma (DePuy, Warsaw, In) and the Triathlon (Stryker, Mahwah, NJ), were included. Only cruciate-retaining (CR) knees in patients who had undergone primary TKA due to primary osteoarthritis (OA) were included to minimise the risk of residual confounding. Revisions for prosthetic joint infections were excluded. At least 2 years of follow-up were required. Surgeries performed by orthopaedic residents were excluded (Fig. 1). The severity of knee OA was assessed from preoperative standing fixed flexion view (FFV) radiographs using the Kellgren–Lawrence (KL) classification. The varus/valgus alignment was assessed from long-leg radiographs. All TKAs were operated to target the mechanical alignment.

Data were obtained from our institution’s Total Joint Replacement Registry. The registry contains prospectively collected information on all joint arthroplasties performed at our institution, including information on patient demographics and all surgery-related preoperative, intraoperative and postoperative follow-up data. The registry receives updates on the possible deaths and emigrations of all patients from the Digital and Population Data Services Agency [4].

The knees were divided into two groups based on the thickness of the PE insert. A “thick PE” was defined as a TKA with a PE insert of 13 mm (mm) or more and a “standard PE” as a TKA with a PE insert of less than 13 mm. This division was based on the standard bony resections used in primary TKA at our institution. The threshold for a thick PE was defined as + 2 size thicker than standard bone resection. The target thicknesses for the PE inserts for each of the knee designs were as follows: the NexGen: 10 mm or 12 mm, the PFC Sigma: 10 mm or 12.5 mm and the Triathlon: 9 mm or 11 mm. These are also the most common PE insert thicknesses used at our institution. The use of thicker inserts at our institution is, however, uncommon, and any PE thickness over 12.5 mm would be considered a “thick” PE insert. Combining this TKA design-specific information, a thickness of 13 mm or more was classified as thick and inserts of less than 13 mm as standard, irrespective of the knee design used.

The primary outcomes were (a) the short-term (< 2 years) and (b) long-term (up to 12.6 years) revision rates of TKAs with any knee revision, except for prosthetic joint infections (PJI), as the endpoint in the analyses. The short-term revision rate was the main area of interest, as early revisions were hypothesised to be directly related to the use of thick PE inserts. Sensitivity analyses were performed for each of the TKA designs separately.

Patient demographics were interpreted as means with standard deviation (SD), as medians with interquartile range (IQR), or as counts with percentages (%), depending on the type and distribution of each variable. The accuracy of the measurements was one decimal.

Kaplan–Meier (KM) survival analysis with 95% confidence intervals (CI) was performed at the 2- and 10-year time points for both thickness groups as a whole and for each insert group separately. Multivariable Cox proportional hazard regression model was used to compare the survival rates between the thickness groups. The Cox model was computed for a) 2-year follow-up and b) for the whole follow-up period. Covariates were selected using directed acyclic graphs (DAG), constructed with online software (dagitty.net) [10] (Appendix 1). Based on the results of the DAG, the included covariates were sex, age, BMI, preoperative malalignment and previous surgery in the multivariable model. Age and BMI were continuous variables and other covariates categorical. Preoperative severe malalignment was more than 10° of varus or valgus in long-leg X-ray. Information from earlier surgeries (i.e., arthroscopy, ligament reconstructions) performed prior to TKA were also reviewed from the data and managed as a logical variable. The thickness of the PE insert (13 mm and more or less than13 mm) was the dependent variable.

Proportional hazard (PH) assumptions were evaluated by the correlation of scaled Schoenfeld residuals with time. PH assumption violation was managed using a time-dependent coefficients method [12]. Proper stratification points were examined from the log–log survival curve visually and added to the Cox model. Schoenfeld residuals were repeatedly checked if non-proportionality was fixed after time stratification.

Statistical analyses were conducted with R software, version 4.0.2 (R Foundation for Statistical Computing, Vienna, Austria), using the packages survival, survminer and tidyverse.

In accordance with Finnish legislation on clinical research, no ethical committee approval or informed written consent was required because of the retrospective register-based study design. Permission to conduct this study was granted by the institutional review board at our institution.

## Results

A total of 7943 primary TKAs were included in the study (Table 1). The NexGen implant comprised 44% of all the TKAs in the study cohort, followed by the PFC Sigma (37%) and the Triathlon (19%).

**Table 1:** Patient demographics

	< 13 (*N* = 6690)	≥ 13 (*N* = 953)	Total (*N* = 7643)
Age			
Mean (SD)	67.8 (8.9)	68.2 (9.3)	67.8 (9.0)
Sex			
Female	4274 (63.9%)	590 (61.9%)	4864 (63.6%)
Male	2416 (36.1%)	363 (38.1%)	2779 (36.4%)
Knee design			
Nexgen	2948 (44.1%)	421 (44.2%)	3369 (44.1%)
PFC Sigma	2561 (38.3%)	279 (29.3%)	2840 (37.2%)
Triathlon	1181 (17.7%)	253 (26.5%)	1434 (18.8%)
Revisions	149 (2.2%)	35 (3.7%)	184 (2.4%)
Nexgen	56 (0.8%)	10 (1.0%)	66 (0.9%)
PFC Sigma	50 (0.7%)	10 (1.0%)	60 (0.8%)
Triathlon	43 (0.6%)	15 (1.6%)	58 (0.8%)
Previous knee surgery	539 (8.1%)	53 (5.6%)	592 (7.7%)
Patellar surfacing	490 (7.3%)	79 (8.3%)	569 (7.4%)
Severe preoperative malalignment^a^	438 (6.5%)	79 (8.3%)	517 (6.8%)
BMI			
Mean (SD)	30.5 (5.3)	31.2 (5.7)	30.6 (5.3)
Years to revision			
Median (Q1, Q3)	1.7 (0.8, 2.7)	1.3 (0.8, 2.2)	1.6 (0.8, 2.7)
Min–Max	0.003–10.7	0.08–7.3	0.003–10.7
Years to death	(*n* = 535)	(*n* = 126)	(*n* = 661)
Median (Q1, Q3)	5.0 (2.7, 7.7)	5.8 (4.0, 7.7)	5.2 (2.8, 7.7)
Min–Max	0.005–12.1	0.03–11.8	0.005–12.1
Follow-up (years)			
Median (Q1, Q3)	4.9 (3.3, 8.6)	7.1 (3.9, 10.4)	5.1 (3.4, 8.9)
Min–Max	2.0–12.6	2.0–12.5	2.0–12.6

^a^Severe malalignment was defined as over 10° of varus or valgus in the long leg X-ray

**Fig. 2:**
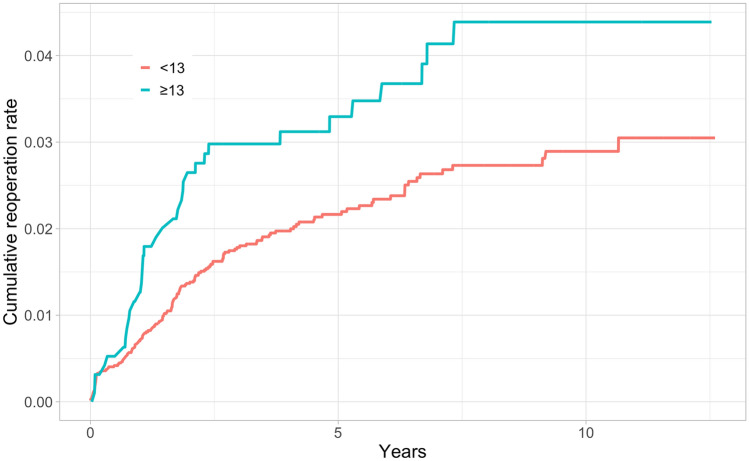
All TKAs: Unadjusted Kaplan–Meier cumulative reoperation rate by thickness of PE insert

**Fig. 3:**
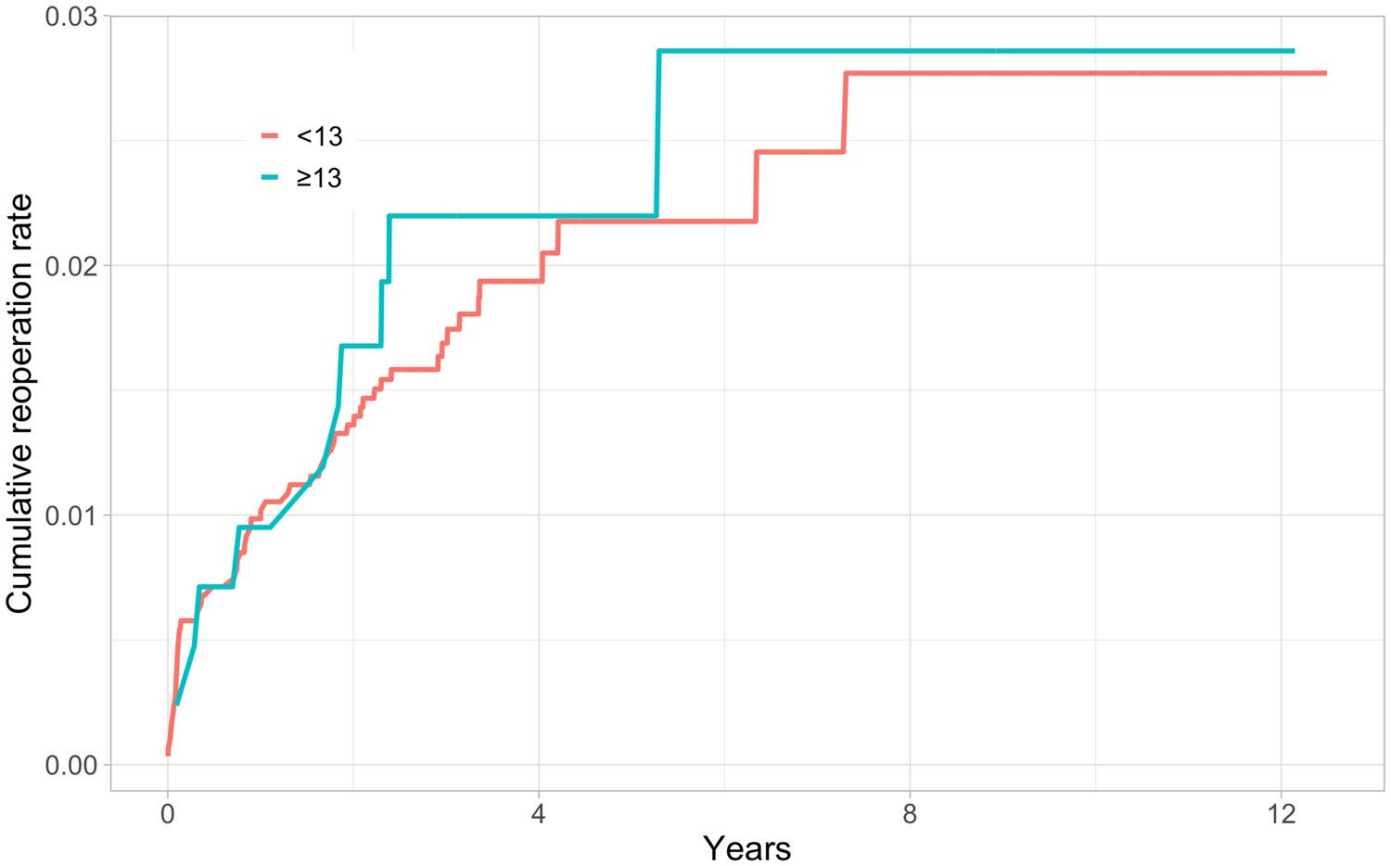
NexGen: Unadjusted Kaplan–Meier cumulative reoperation rate by thickness of PE insert

**Fig. 4:**
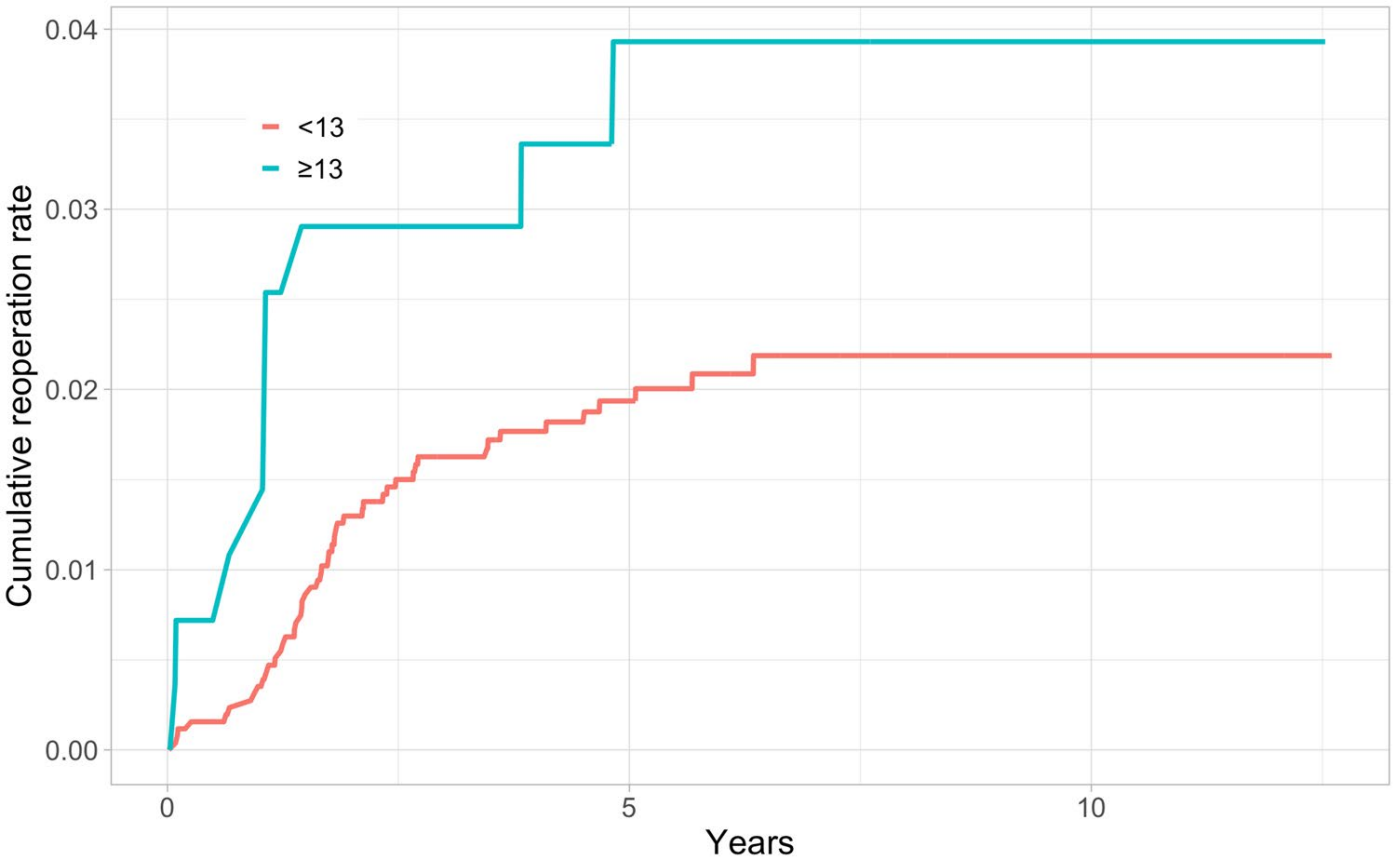
PFC Sigma: Unadjusted Kaplan–Meier cumulative reoperation rate by thickness of PE insert

**Fig. 5:**
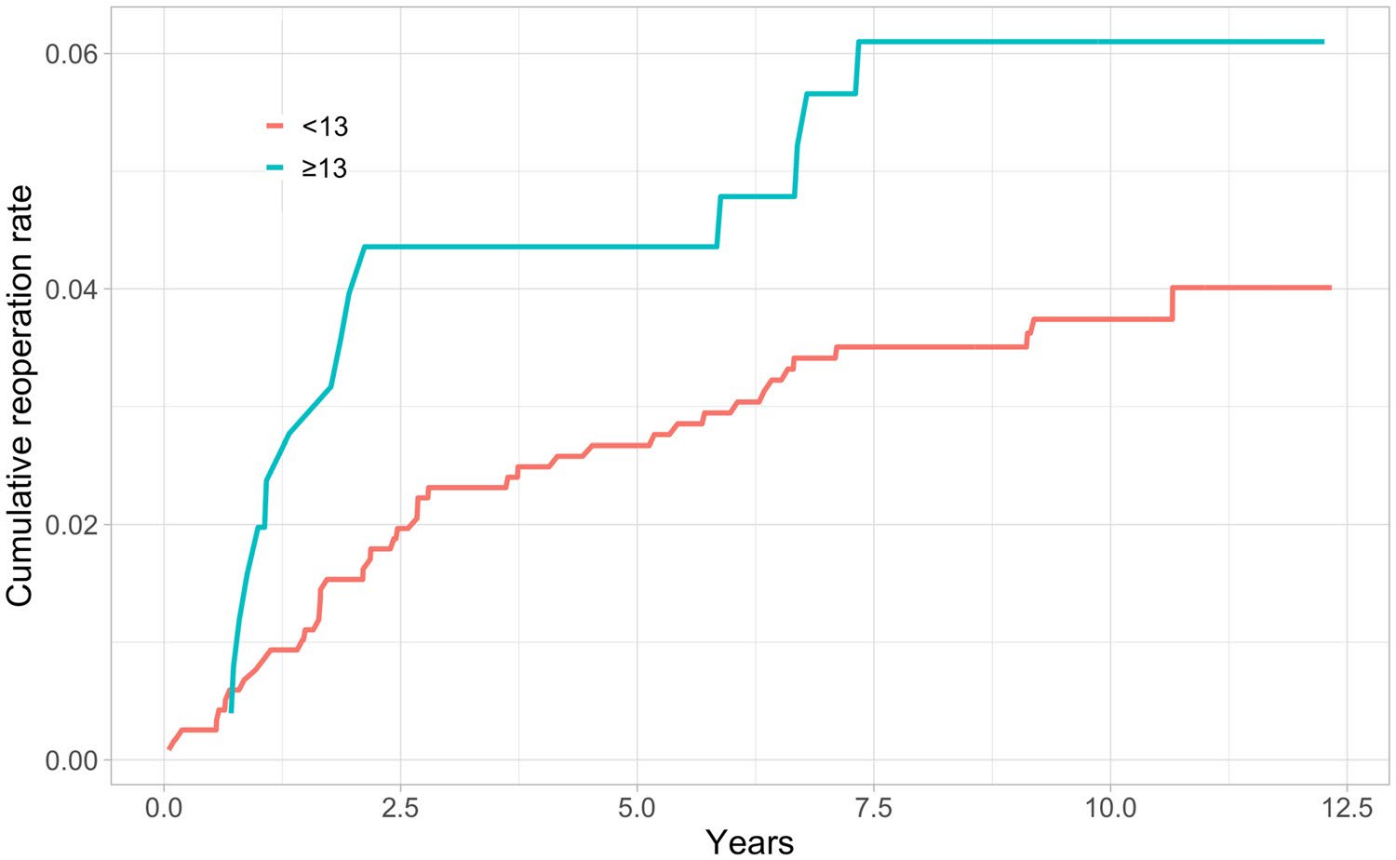
Triathlon: Unadjusted Kaplan–Meier cumulative reoperation rate by thickness of PE insert

In total, 184 (2.4%) of the knees in the study cohort underwent revision surgery during the whole 12.6-year follow-up, and 116 (63%) were performed within the first 2 years after primary TKA. Kaplan–Meier 2-year revision rates were higher for the thicker PE group in both the short- and long-term (Table 2) (Figs. 2, 3, 4, 5).

**Table 2:** Kaplan–Meyer (KM) revision rates for 2- and 10-year time points with 95% confidence intervals (CI)

Group	Thickness of PE	N of TKAs	N of revisions	At risk at 2 years	KM-revision rate (%) at 2 years	At risk at 10 years	KM-revision rate (%) at 10 years
All	< 13	6690	149	6508	1.4 (1.1–1.6)	882	2.9 (2.4–3.4)
	≥ 13	953	35	911	2.6 (1.6–3.7)	211	4.4 (2.9–5.9)
NexGen	< 13	2948	56	2874	1.4 (0.9–1.8)	107	2.8 (1.7–3.8)
	≥ 13	421	10	405	1.7 (0.4–2.9)	41	2.9 (0.9–4.8)
PFC Sigma	< 13	2561	50	2490	1.3 (0.9–1.7)	212	2.2 (1.6–2.8)
	≥ 13	279	10	264	2.9 (0.9–4.9)	31	3.9 (1.5–6.3)
Triathlon	< 13	1181	43	1144	1.5 (0.8–2.2)	563	3.7 (2.6–4.8)
	≥ 13	253	15	242	4.4 (1.8–6.8)	139	6.1 (3.1–9.1)

In the Cox regression model, the thicker PE insert group also evinced an increased risk for revision both in short-term (< 2 years) follow-up and during the whole follow-up period compared to the standard PE insert group. In the PFC Sigma subgroup, non-proportionality had to be fixed by stratification of the short-term follow-up due to the time-dependent coefficients method used in the Cox regression model. Thus, short-term follow-up was divided into 0 to 0.7 and 0.7 to 2.0 years. The sensitivity analysis between the different implant design groups revealed that the risk was mainly observed in the Triathlon group short term and in the PFC Sigma group during the 0 to 0.7-year period (Table 3).

**Table 3:** Multivariate Cox regression models with adjusted hazard ratios and 95% confidence intervals

	Thickness of the PE bearing	Time group (years)	HR	95% CI
ALL^a^	13 mm and over	0–13	1.6	1.1–2.3
	13 mm and over	0–2	2.0	1.3–3.2
Nexgen^b^	13 mm and over	0–13	1.1	0.6–2.2
	13 mm and over	0–2	1.2	0.5–2.6
PFC Sigma^c^	13 mm and over	0–0.7	5.6	1.4–25.0
	13 mm and over	0.7–2.0	2.2	0.9–5.9
	13 mm and over	2.0–12.6	0.7	0.09–5.0
Triathlon^d^	13 mm and over	0–13	1.7	0.9–3.0
		0–2	2.6	1.2–5.7

Adjusted by age, sex, BMI, surfacing of patella, previous operation, and severity of preoperative malalignment. Time-dependent coefficients divided into time intervals as follows:

^a^2-year analysis: sex and preoperative malalignment into 0.3

^b^Sex into 0.03, 0.6 and 1.1 years and for 2-year analysis sex 0.3 years

^c^Thickness of the PE insert and age into 0.7 and 2 years

^d^2-year analysis: Sex into 0.8 years

## Discussion

The most important finding of the present study was the markedly increased revision rate in primary TKAs with thicker polyethylene inserts. Sensitivity analyses showed that the difference in revision rates was most remarkable in favour of standard PE inserts with Triathlon TKAs. This difference had already been identified in short-term follow-up, and the divergent trend between the survival curves continued throughout the whole follow-up period. The Triathlon TKA was used at our institution until 2013, and the reason for this finding is unclear, as similar surgical techniques were used to balance the knee intraoperatively, irrespective of the TKA design used. The Triathlon TKA has a single-radius design which differs from the PFC Sigma and NexGen designs. Therefore, it may be more sensitive for revision with thicker inserts. There was also a higher revision rate identified in the PFC Sigma subgroup very short term. In the NexGen and the other PFC Sigma time-period subgroups, there was a slight trend for higher revision rates in knees with thicker PE inserts. In the Cox regression model, the 2-year analysis of the PFC Sigma subgroup was divided into time periods of 0 to 0.7 and 0.7 to 2 years because of the non-proportionality of Schoenfeld residuals.

To date, there are only three previous studies that have investigated the effect of PE insert thickness on implant survival. Berend et al. [2] found that thicker PE (16 mm or more) inserts were associated with higher failure rates in TKA. In their study, they included 5997 AGC (Biomet) CR TKAs and used similar inclusion criteria as in the present study. A total of 53 TKAs underwent revision due to mechanical issues during a mean follow-up time of 6.8 years. For thicker inserts, the likelihood ratio for failure was 3.2 times higher and survivorship was 4% lower [2].

Namba et al. [8] investigated the effect of PE insert thickness (15 mm or more) on the outcomes of primary TKA in high-flexion and non-high-flexion TKA designs from different implant manufacturers. They included 64 000 TKAs with both CR- and posterior-stabilized (PS) designs. The mean follow-up was 3.3 years. In their study, the highest revision risk was discovered with the NexGen high-flexion fixed CR design with HR of 9.08 (95% CI 1.58 to 52.32). However, there were only two revisions in the thicker PE insert group and the sample sizes remained relatively small in each subgroup. The non-high-flexion NexGen components did not demonstrate an increased risk for revision with thicker tibial inserts [8].

In contrast, Greco et al. [4] studied clinical outcomes, revision rates and overall implant survival rates for 6698 Vanguard TKAs, where the PE insert was defined as thick when it was 15 mm or more. Consequently, 3.5% of the inserts were considered thick. In their study, the type of PE insert varied, as the study included both standard CR PE inserts, posterior lip inserts and highly conforming anterior-stabilised inserts with significant posterior build-ups. Mean follow-up was 5.6 years. Revision and implant survival rates were similar between groups, and no failures for aseptic loosening or instability were detected. However, there were four revisions in the thick PE group and two of these were due to prosthetic joint infection [4].

It must be noted that in these earlier studies a thick PE insert was defined as between 15 and 16 mm or more, whereas in the present study, a thick PE insert was defined as 13 mm or more. Our rationale for choosing 13 mm as the borderline thickness in this study derives directly from clinical practice. Regardless of the TKA design used, the aim is a polyethylene thickness of between 9 and 10 mm with the bone cuts. In our experience, most surgeons who perform primary TKAs with contemporary designs follow a rather similar surgical strategy. Thus, if one aims at a 9–10 mm polyethylene insert with the tibial cut, and ends up using a 11–12 mm insert, it is still a very small difference clinically. With similar bony cuts, one knee may require a 10 mm PE insert, whereas the other knee may require a 12 mm PE insert to achieve correct ligamentous stability. This is because there are individual differences in the soft tissue tension around the knee. Moreover, there is also variability in the amount of ligamentous stability that different surgeons are willing to accept intraoperatively. Thus, a 2 mm difference in the planned vs chosen polyethylene thickness seldom indicates postoperative problems. However, from a clinical point of view, if one aims at a polyethylene insert thickness of 9–10 mm, but ends up using 13–14 mm or even more, there is usually something abnormal in the knee. This abnormality may be caused by inherited ligamentous laxity, deep tibial resection, gap imbalance or iatrogenic collateral ligament injury [2].

Clinical problems caused using a thicker PE insert are, however, rare. Indeed, as reported in the current study, knees with a thicker PE insert still showed survival rates of over 90% at long-term follow-up, regardless of the TKA design used. Still, the revision risk markedly increased with the use of thicker PE inserts. This finding may indicate that in a small proportion of these knees the surgeon has tried to solve an intraoperative problem using a thick PE insert. Unfortunately, in some cases, increasing the thickness of the PE insert is not the right solution and the intraoperative change to a more constrained TKA design would most probably have been warranted. If a thick PE insert is required to satisfactorily stabilize the knee after the standard bony cuts and ligamentous balancing, one should carefully assess the reasons for this and consider using a more constrained TKA design if there is still something abnormal in ligamentous stability, knee range of motion or gap balance in the knee. Using a constrained TKA design, the stability of the TKA is achieved, and it no longer relies solely on the balance of the ligaments. As a result, the outcome is expected to be better than using a CR component.

We acknowledge a few limitations in the present study. First, we applied different cut points for the “standard” and the “thick” groups compared to previous studies, and this complicated the direct comparison of our results to the earlier literature. However, when the difference in revision rates between the PE insert thickness groups is observed with a lower cut point, it supports the hypothesis that thicker PE inserts lead to a greater risk for revisions. Second, the sample sizes in our TKA-specific subgroups were relatively small, and there were only 35 revisions in the thicker PE insert group. We did not compare the different implant types, for example, CR vs more constrained designs. This might have provided more information on whether the more constrained designs would have had lower revision rates. Furthermore, the reasons for the revisions could have been assessed in more detail.

We also feel the current study has some strengths that serve to increase the generalisability of the results. First, we had a large patient cohort with complete follow-up data from a single high-volume joint replacement centre. Second, our data contain different implant designs and an individual analysis for each of these designs was performed. Third, we only included the non-constrained type of CR designs to minimise residual confounding. In the study by Greco et al. [4], variable PE insert bearing types were included. However, when the bearing types are unevenly distributed in the study groups, it may be the bearing type rather than the thickness of the PE insert that explains the possible differences observed in revision rates.

## Conclusion

We found a higher revision rate in those TKAs in which thicker PE inserts were used. The highest risk for revision was found in the thick PE inserts used with the Triathlon TKA. The differences were mainly detected during the first 2 years after the primary operation. Therefore, when an unusually thick PE bearing is required in primary TKA, the surgeon should always carefully assess the reason for this and consider converting the surgery into a more constrained TKA design.

## Supplementary Information

Below is the link to the electronic supplementary material.

Additional file 1. Directed acyclic graph representing the causal relationships behind the multivariable Cox regression model. (PDF 194 KB)

## Abbreviations

BMI: Body-mass index
CI: Confidence interval
CR: Cruciate-retaining
DAG: Directed acyclic graphs
FAR: Finnish Arthroplasty Register
HR: Hazard ratio
IQR: Interquartile range
KM: Kaplan–Meier
OA: Osteoarthritis
PE: Polyethylene
PH: Proportional hazard
PJI: Prosthetic joint infection
PS: Posterior-stabilised
SD: Standard deviation
TKA: Total knee arthroplasty
