# Supplementary figures and images for: Thicker polyethylene inserts (≥ 13 mm) increase the risk for early failure after primary cruciate-retaining total knee arthroplasty (TKA): a single-centre study of 7643 TKAs
Source: Knee Surg Sports Traumatol Arthrosc. 2022 Oct 7;31(3):1018–25. doi: 10.1007/s00167-022-07189-8 (PMC9957842; doi:10.1007/s00167-022-07189-8)

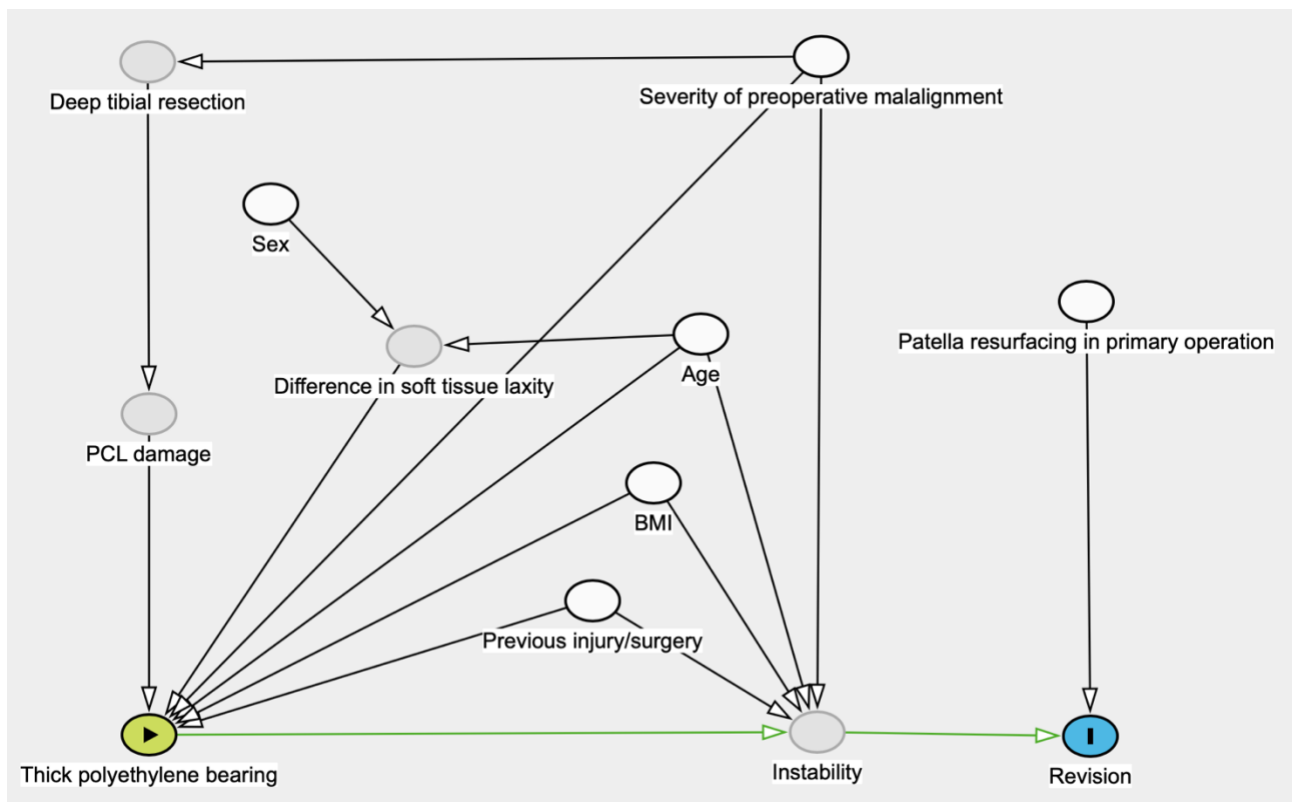

Supplement: Supplementary file 1 — Additional file 1. Directed acyclic graph representing the causal relationships behind the multivariable Cox regression model. (PDF 194 KB) [file 167_2022_7189_MOESM1_ESM.pdf]
